# Standing Genetic Variation in Contingency Loci Drives the Rapid Adaptation of *Campylobacter jejuni* to a Novel Host

**DOI:** 10.1371/journal.pone.0016399

**Published:** 2011-01-24

**Authors:** John P. Jerome, Julia A. Bell, Anne E. Plovanich-Jones, Jeffrey E. Barrick, C. Titus Brown, Linda S. Mansfield

**Affiliations:** 1 Comparative Enteric Diseases Laboratory, Michigan State University, East Lansing, Michigan, United States of America; 2 Department of Microbiology and Molecular Genetics, Michigan State University, East Lansing, Michigan, United States of America; 3 Department of Computer Science and Engineering, Michigan State University, East Lansing, Michigan, United States of America; 4 Department of Chemistry and Biochemistry, Institute for Cell and Molecular Biology, The University of Texas at Austin, Austin, Texas, United States of America; Charité-University Medicine Berlin, Germany

## Abstract

The genome of the food-borne pathogen *Campylobacter jejuni* contains multiple highly mutable sites, or contingency loci. It has been suggested that standing variation at these loci is a mechanism for rapid adaptation to a novel environment, but this phenomenon has not been shown experimentally. In previous work we showed that the virulence of *C. jejuni* NCTC11168 increased after serial passage through a C57BL/6 IL-10^-/-^ mouse model of campylobacteriosis. Here we sought to determine the genetic basis of this adaptation during passage. Re-sequencing of the 1.64Mb genome to 200-500X coverage allowed us to define variation in 23 contingency loci to an unprecedented depth both before and after *in vivo* adaptation. Mutations in the mouse-adapted *C. jejuni* were largely restricted to the homopolymeric tracts of thirteen contingency loci. These changes cause significant alterations in open reading frames of genes in surface structure biosynthesis loci and in genes with only putative functions. Several loci with open reading frame changes also had altered transcript abundance. The increase in specific phases of contingency loci during *in vivo* passage of *C. jejuni*, coupled with the observed virulence increase and the lack of other types of genetic changes, is the first experimental evidence that these variable regions play a significant role in *C. jejuni* adaptation and virulence in a novel host.

## Introduction

*Campylobacter jejuni* is a leading cause of bacterial gastroenteritis in humans and is a common antecedent infection to the acute flaccid paralysis, Guillain-Barré syndrome [1], [2]. It is considered a commensal of avian species, and most human infections likely arise from contact with raw chicken meat [3]. However, *C. jejuni* has a large natural host range including cows, pigs, dogs, cats, migratory birds, and insects [4], [5], [6], [7], [8]. Despite the ability to colonize a diverse range of hosts there has been a lack of small animal models for study of human disease, and little understanding of the molecular basis of *C. jejuni* virulence.

When the genome of *C. jejuni* NCTC11168 was sequenced in 2000 it was found to contain multiple hypervariable regions, or contingency loci, that could not be resolved to a consensus sequence [9]. Contingency loci are important for virulence properties in other pathogenic bacteria, including *Haemophilus influenzae*, *Neisseria gonorrhoeae*, and *Helicobacter pylori* [10], [11], [12]. In *C. jejuni* these regions consist of homopolymeric tracts of nucleotides that are prone to slipped-strand mispairing during replication [13]. This leads to a high rate of indel mutation that can change the open reading frame of a gene. These mutations are a mechanism for phase variation, as they often control ON/OFF phenotypic switches. Contingency loci are thought to be a bet-hedging strategy in case of exposure to novel situations, since cells derived from a single ancestral cell can have numerous heritable phenotypes [14]. To test whether these genes are important for rapid adaptation in *C. jejuni*, Wassenaar *et al.* attempted to determine patterns of variation in six contingency loci during experimental adaptation [15]. In response to heat and cold stress, passage through tissue culture cells, and passage through the chicken gut, the distribution of variation across the six sites under investigation was stable; providing no evidence for the role of contingency genes in adaptation to a novel environment. However, it is possible that contingency genes not investigated had mutated or that the conditions tested were not selective for specific contingency gene phases.

Serial passage experiments are a method to increase pathogen virulence by adaptation to a specific host [16]. This experimental evolution involves host infection, followed by re-isolation of the pathogen, and then inoculation of the re-isolated pathogen into a new host. Serial passage of viral, bacterial, and eukaryotic pathogens almost exclusively results in increased fitness, virulence, and growth rate in the host, and decreased virulence to a former host [16], [17]. Comparative sequence analysis of the adaptation that occurs in such experiments has only been performed for viruses. For example, Brown *et al.* re-sequenced the viral genome of influenza A after experimentally increasing virulence in the mouse lung [18]. In this case, serial passage followed by genome re-sequencing led to the discovery of multiple novel virulence modulators. Studies like this show that serial passage leading to increased virulence followed by the analysis of genetic mutations can be used as a forward genetic screen for virulence factors.

*In vivo* serial passage of *C. jejuni* alters virulence. Jones *et al.* showed that passage of a poorly motile variant of *C. jejuni* through chickens resulted in restored motility and increased ability to colonize and persist in the avian gastrointestinal tract [19]. A reduction in the minimum infectious dose of *C. jejuni* has also been reported as a result of passage through chickens [20]. Prior work described by Bell *et al.* showed that when *C. jejuni* was serially passaged through the cecum of C57BL/6 IL-10^−/−^ mice, the virulence of mouse-adapted bacteria increased [21]. Higher measures of pathology that included the presence of blood in the cecal lumen, decreased timing of the onset of disease, and higher fecal and jejunal *C. jejuni* population sizes indicated that the mouse-adapted bacterial population was more virulent than the ancestral *C. jejuni*. Increased virulence following serial passage was observed for three of five *C. jejuni* strains tested, and higher virulence could often be observed in bacteria re-isolated after a single 35-day *in vivo* passage. These results suggest that adaptation occurs rapidly during the course of *C. jejuni* infection.

Our objective in this study was to test the hypothesis that specific genetic changes in *C. jejuni* control adaptation to a novel host. The capacity to increase virulence by *in vivo* passage is well documented, and uncovering mutations on the genome-scale is now feasible using new sequencing technologies [22], [23], [24], [25]. We used Illumina sequencing to re-sequence the genome of *C. jejuni* NCTC11168 before (referred to as wild-type) and after mouse serial passage (referred to as mouse-adapted), as well as expression microarrays, pulse-field gel electrophoresis, and phenotypic assays to determine the basis of *C. jejuni* host adaptation during passage. We found that variation present in contingency loci in the ancestral inoculum is driving the adaptation of *C. jejuni* during *in vivo* serial passage. Deep re-sequencing showed that the frequencies of specific phases at contingency loci changed during passage. Furthermore, several contingency loci with sequence changes also had altered transcript abundance based on microarray and real-time qRT-PCR. Many contingency genes that mutated during passage are putatively involved in surface carbohydrate biosynthesis, and as expected, phenotypic assays that are affected by surface structure changes in *C. jejuni* revealed differences between the mouse-adapted and wild-type variants.

## Results

### Deep re-sequencing of contingency loci variation

We suspected that variations in the 8–13 base homopolymeric tracts of contingency loci would play a role in *C. jejuni* adaptation during serial passage. However, the utility of next-generation sequencing technologies in evaluating variations in contingency loci had not been reported. We chose to re-sequence the NCTC11168 genome using Illumina short-read technology for two reasons. First, this platform provides very high coverage, which is necessary to observe frequencies of indels in homopolymeric tracts. Second, in contrast to pyrosequencing, Illumina sequencing is not known to suffer from a higher than average error rate for indels in homopolymeric DNA. One channel of an Illumina Genome Analyzer II flow cell was used per variant to generate sequence coverage of approximately 200-500X across the genome. Manual curation of reads mapping to homopolymeric tracts yielded an average coverage of 107X per tract, with a range of 42-252X, for reads that unambiguously defined the number of bases within the homopolymeric tract. Additionally, the estimated error rate for indel mutations in this data set was lower than the estimated base substitution error rate (Figure S1), as has been shown by others [26], suggesting that indel mutation analysis is more robust to sequencing errors than SNP discovery. When we characterized homopolymeric tracts by colony PCR and Sanger sequencing, the results were consistent with the Illumina short read sequence data, further verifying the use of this technology for re-sequencing the variable tracts of contingency loci in *C. jejuni*.

### Variations in contingency genes after *in vivo* passage

**Figure 1:**
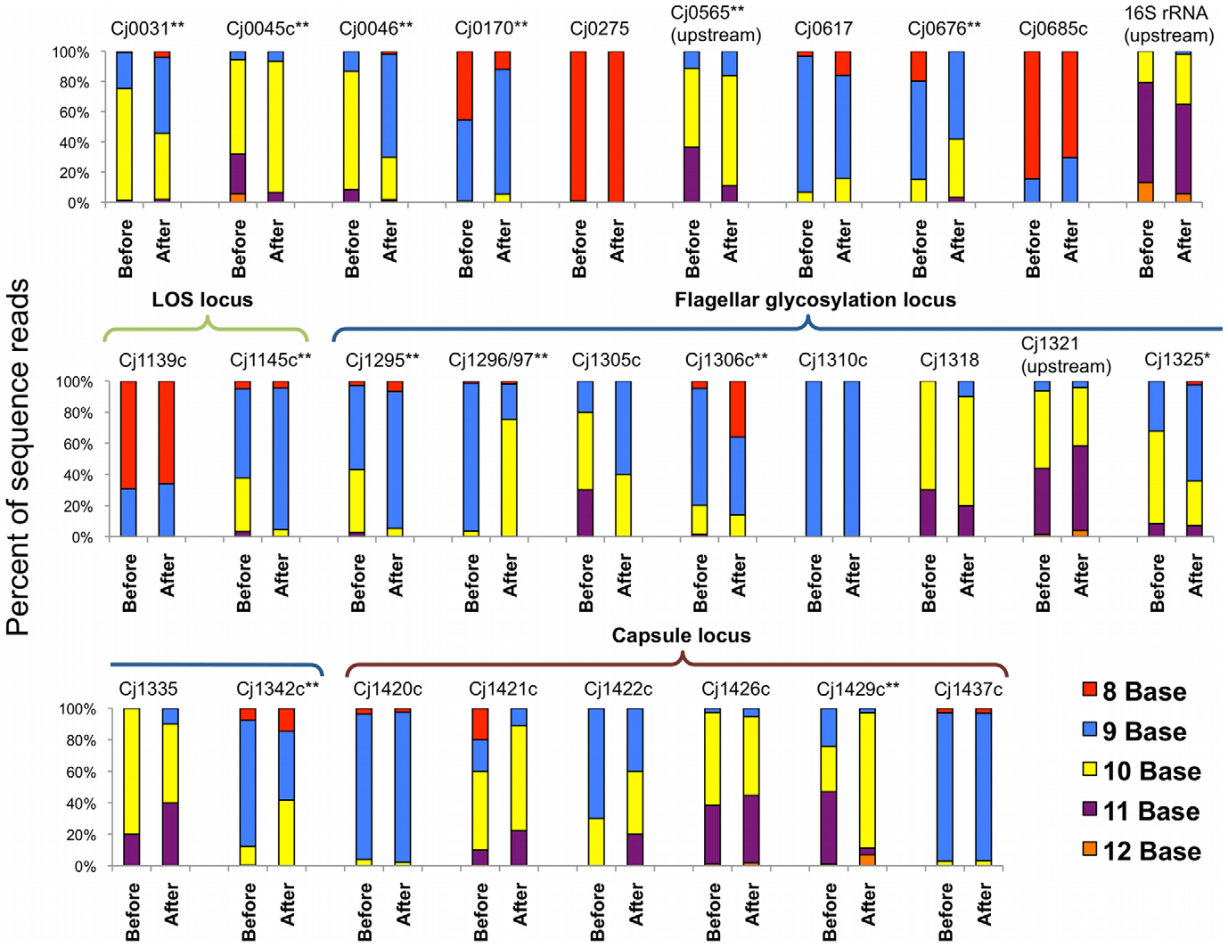
Microevolution of contingency loci. For each contingency gene, the percentage of sequence reads with a particular base count in the homopolymeric tract is graphed. The distribution of homopolymeric variations before (wild-type *C. jejuni* NCTC11168), and after (mouse-adapted *C. jejuni* NCTC11168) passage, for each variable gene in the genome is shown. *Cj0628* contains 2 variable tracts, but is not pictured. The distribution of variation at this tract was stable through passage. * p≤0.01 and ** p≤0.001 by chi-square test of distribution for open reading frame variations.

Twenty-three variable homopolymeric tracts, and nine potentially variable tracts, were reported when the NCTC11168 genome was sequenced in 2000 [9]. In our re-sequencing analysis 28 homopolymeric tracts were variable. We found no indel mutations for *Cj1367c*, containing a reportedly variable two base guanine tract, and *Cj1677*, with a 7 base thymine tract. *Cj0628* contains two adjacent homopolymeric tracts, and both were variable. The homopolymeric thymine tract in *Cj0628* was the only variable thymine tract found, despite the presence of numerous long thymine tracts in the AT-rich *C. jejuni* genome. Because sequences surrounding six tracts (*Cj1305c*, *Cj1310c*, *Cj1318*, *Cj1335*, *Cj1421c*, and *Cj1422c*) match multiple sites in the genome, short sequence reads could not be unambiguously mapped to these loci. These tracts were defined by colony PCR and Sanger sequencing as described in the Methods section. Every homopolymeric tract containing at least 8 guanines or cytosines in the NCTC11168 genome was variable with the exception of the 9 base tract in *Cj1310c*. However, this tract only had coverage of 10X by colony PCR and Sanger sequencing. Variations in the entire suite of NCTC11168 contingency loci before and after mouse-adaptation are shown in Figure 1.

For several contingency loci a specific phase increased in frequency during passage. As an example, in *Cj1296*, 3.6% of sequence reads had 10 guanine residues before passage, and after passage 75.5% of reads showed 10 guanines. Insertion of a guanine at this site creates a fusion of open reading frames *Cj1296* and *Cj1297*. For other contingency loci such as *Cj1139c* and *Cj1420c* the distribution of indel variants was stable through passage. Overall, chi-square tests of association revealed significant differences at thirteen homopolymeric tracts as a result of mouse adaptation (Table 1). All of these differences are associated with open reading frame changes, except for the tract at *Cj0565,* which is upstream of the potential ORF. Both positive and negative in-frame ORF fold changes can be seen in Table 1. Therefore, the homopolymeric tract length corresponding to the in-frame ORF was enriched due to mouse adaptation for some contingency loci (*Cj0031*, *Cj0046, Cj0676*, *Cj1295*, *Cj1296/Cj1297*, *Cj1325*, and *Cj1429c*), while for others there appears to be selection for an ORF with a premature stop codon.

**Table 1:** Significant open-reading frame changes due to serial passage.

Gene	Putative Function	Variations Detected	In-frame ORF tract length^a^	Enriched ORF	Percent with enriched ORF		Fold change of in-frame ORF^c^
					Wild-type	Mouse-adapted	
*Cj0031*	Restriction/Modification	G(8-11)	G(9)	In-frame	24.0	50.5	2.1
*Cj0045c*	Iron-binding protein	C(9-13)	C(11)	Frameshifted	74.0	93.5	−4.0
*Cj0046*	Pseudogene	G(8-13)	G(9)	In-frame	13.2	68.7	5.2
*Cj0170*	Conserved hypothetical	G(8-10)	G(8)	Frameshifted	54.6	88.0	−3.8
*Cj0565*	Pseudogene	G(9-11)	None apparent	G(10)^b^	52.3	72.8	
*Cj0676*	Pseudogene	G(8-11)	G(10)	In-frame	15.1	38.7	2.6
*Cj1145c*	Glycosyltransferase	C(8-11)	C(10)	Frameshifted	65.6	95.5	−7.6
*Cj1295*	Aminopeptidase	G(6-11)	G(9)	In-frame	54.2	86.8	1.6
*Cj1296/Cj1297*	Aminoglycoside N3’-acetyltransferase	G(8-10)	G(10)	In-frame	3.6	75.5	20.9
*Cj1306c*	Unknown	C(8-11)	C(9)	Frameshifted	25.0	50.0	−1.5
*Cj1325*	Acetimidino-N-methyltransferase	G(8-11)	G(9)	In-frame	32.2	61.9	1.9
*Cj1342c*	Unknown	C(8-11)	C(9)	Frameshifted	19.9	56.3	−1.8
*Cj1429c*	Unknown	C(9-13)	C(10)	In-frame	28.9	86.1	3.0

a The “In-frame ORF tract length” was defined as the most frequently observed homopolymeric tract length that generated the longest potential open-reading frame. All tract lengths that introduced a premature stop codon were considered frameshifted.

b A tract with length 10 increases in frequency during passage, but no ORF change could be detected since the tract is upstream of the predicted coding region.

c The tract lengths observed for *Cj0046*, *Cj1295*, and *Cj1429c* each contained a single read that differed from the “In-frame ORF tract length” by 3 guanines (or cytosines), and these were considered in-frame since they do not introduce a premature stop codon. A positive fold change indicates an increase of the in-frame ORF in mouse-adapted *C. jejuni*, and a negative fold change indicates a decrease.

Five significant changes in contingency genes were in the flagellar glycosylation locus, and there was a single change in each of the lipooligosaccharide (*Cj1145c*) and capsule biosynthesis (*Cj1429c*) loci. Several contingency genes annotated as pseudogenes (*Cj0046*, *Cj0565*, *Cj0676*) and a conserved hypothetical protein (*Cj0170*) also changed significantly. Altered open reading frames of contingency genes annotated as a restriction/modification enzyme (*Cj0031*) and an iron-binding protein (*Cj0045c*) were also enriched after passage.

### Mutations outside of contingency genes

Single-nucleotide polymorphisms (SNPs) along with duplications, insertions, and other polymorphisms (DIPs) have been reported as the dominant genetic basis of adaptation in evolution experiments with microbes [18], [23], [24]. We analyzed the Illumina short reads for the presence of SNPs and DIPs. There was no evidence of DIPs, and only one SNP was found to change in frequency by more than 20% during passage. This is a nonsynonymous mutation that changes a valine to alanine in *pbpC* (V30A), and was confirmed by Sanger sequencing. This mutation arose during passage and increased in frequency to approximately 62% after three *in vivo* passages. Six other SNPs were predicted to have changed in frequency during passage by at least 5%, but less than 16% (Table S1). The absence of fixed SNPs and DIPs during passage was not a result of the sequencing methods or analysis, as multiple fixed SNPs and DIPs were found in the genome of the NCTC11168 (ATCC 700819) culture we used compared to the NCTC11168 that was sequenced in 2000 (Table S2). Differences in phenotype between NCTC11168 isolates have been described [27], so genetic differences in our stock culture were not unexpected, and will be informative for future work.

### Absence of large genomic rearrangements

**Figure 2:**
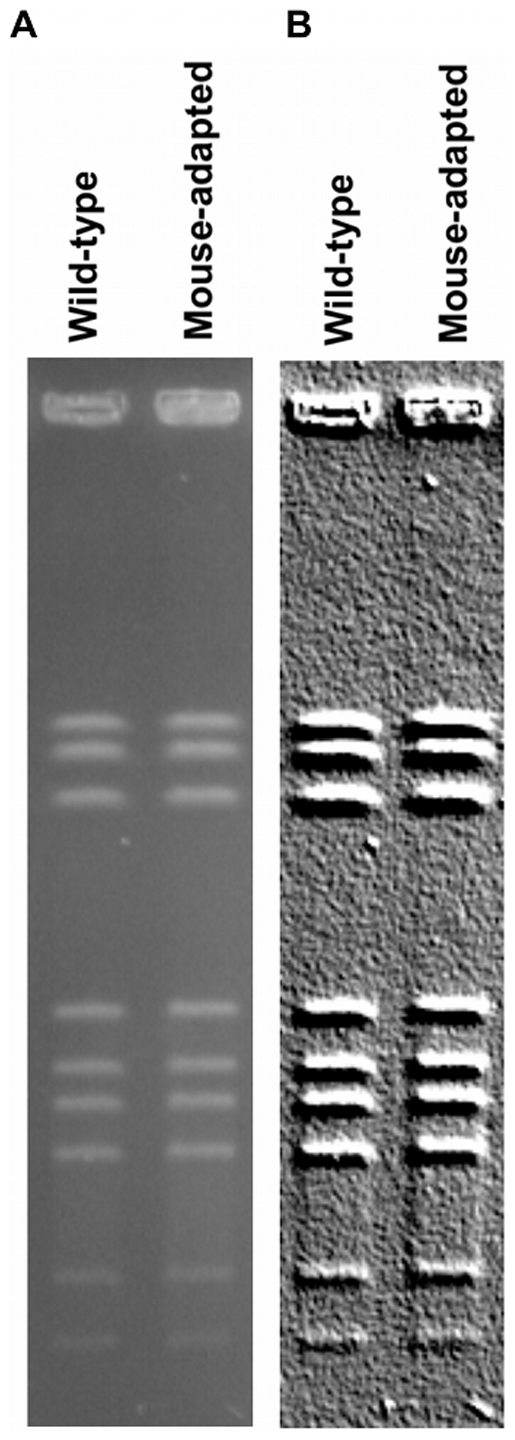
Absence of large genomic changes during passage. (A) Pulse field gel electrophoresis image to compare wild-type and mouse-adapted bacteria. (B) The same gel with an image filter as an attempt to detect rare bands.

Researchers have described large intra-genomic inversions in *C. jejuni* during avian colonization [28], [29] and in particular, in response to bacteriophage predation [30]. Since certain large genomic rearrangements are difficult or impossible to discover using short read re-sequencing technology, we performed pulse-field gel electrophoresis (PFGE) on the wild-type and mouse-adapted *C. jejuni*. PFGE showed no evidence of genome rearrangements in mouse-adapted *C. jejuni* (Figure 2).

### *In vitro* gene expression: wild-type versus mouse-adapted variants

Expression microarrays were used to determine whether adaptation to increased virulence had a basis in transcriptional regulation. From direct comparisons of RNA isolated during growth *in vitro*, nine ORFs were found to have significantly altered expression in the mouse-adapted bacteria relative to wild-type (Table 2). Of these, the five ORFs with the highest fold changes in expression were associated with contingency loci (*Cj0170*, *Cj0565*, *Cj1295*, *Cj1296*, and *Cj1297*). As shown above, these loci also had significant changes in homopolymeric tract length. The homopolymeric tract is located immediately upstream of the ORF predicted for *Cj0565*, but the tract is within the predicted ORFs of *Cj0170*, *Cj1295*, and *Cj1296*. Alteration in transcript abundance was verified for four of these ORFs by real-time qRT-PCR (Table 2). Changes observed by microarray could not be verified for *Cj1190c* and *Cj1381*, which are not contingency loci. Notably, the five contingency loci with altered expression after three passages in mice also showed significant changes by microarray in *C. jejuni* isolated after a single passage (data not shown).

**Table 2:** Genes differentially expressed in the mouse-adapted variant during *in vitro* growth.

Gene	Contingency locus	P-value	Fold Change	
			Microarray	qRT-PCR
*Cj1297*	Yes	0.004	2.5	3.5
*Cj1296*	Yes	0.004	2.1	2.6
*Cj1295*	Yes	0.131	1.6	2.5
*Cj0565*	Yes	0.015	−2.1	NT^a^
*Cj0170*	Yes	0.015	−1.8	−18.9
*Cj0299*	No	0.015	1.8	NT
*Cj1190c*	No	0.016	−1.7	NV^b^
*Cj1021c*	No	0.036	1.6	NT
*Cj1381*	No	0.039	1.6	NV

a NT – not tested.

b NV – tested, but not verified.

### *In vivo* gene expression: wild-type versus mouse-adapted variants

Since many *C. jejuni* genes are specifically regulated *in vivo* [31], we sought to compare transcript abundance of wild-type and mouse-adapted bacteria during mouse infection. To avoid isolation of RNA from commensal microflora, C57BL/6 IL-10^−/−^ germ-free mice were infected with wild-type or mouse-adapted *C. jejuni*, and a protocol to isolate intact, bacterial RNA, free of eukaryotic-RNA contamination, from the cecum was developed (Methods). Four germ-free mice were inoculated with each variant and infection was allowed to proceed for four days. In this short infection period, both variants caused only mild pathology which is consistent with the results of acute *C. jejuni* infection of specific-pathogen-free mice [32], and germ-free mice [33]. At necropsy all mice were colonized only with *C. jejuni*. RNA was obtained from cecal samples and used to directly compare wild-type and mouse-adapted gene expression during *in vivo* growth. This comparison yielded nine significant expression differences between variants (Table 3). Contingency loci *Cj0170*, *Cj0565*, and *Cj1297* were among those with significant transcript abundance changes at p<0.05, while *Cj1296* was significant at p<0.10 by microarray. As described above, these contingency genes also had altered expression *in vitro* and the transcript abundance differences were confirmed by real-time qRT-PCR. However, gene expression differences could not be verified by real-time qRT-PCR for *Cj1506c*, *Cj1523c*, *Cj0144*, and *frdC*, none of which are contingency loci.

**Table 3:** Genes differentially expressed in the mouse-adapted variant during *in vivo* growth.

Gene	Contingency locus	P-value	Fold Change	
			Microarray	qRT-PCR
*Cj1523c*	No	<0.001	2.1	NV^b^
*frdC*	No	<0.001	1.8	NV
*Cj1297*	Yes	<0.001	1.7	2.6
*Cj0565*	Yes	<0.001	−1.6	−2.0
*rpmB*	No	0.003	−1.5	NV
*Cj1506c*	No	<0.001	−1.4	NV
*Cj0170*	Yes	0.027	−1.4	−3.0
*Cj0704*	No	0.027	−1.4	NT^a^
*Cj0144*	No	0.046	−1.4	NT

a NT – not tested.

b NV – tested, but not verified.

### *In vitro* phenotypes of *C. jejuni* variants

The majority of contingency genes are coincident on the genome with surface carbohydrate biosynthetic loci [9], and seven of the thirteen significant contingency gene changes in mouse-adapted bacteria were in these loci. Genes in the flagellar glycosylation locus are known to affect motility and autoagglutination [34], and lipooligosaccharide and capsular biosynthesis genes are known to affect the interaction of *C. jejuni* with epithelial cells in culture [35], [36]. We investigated possible changes in these phenotypes in mouse-adapted *C. jejuni*.

**Figure 3:**
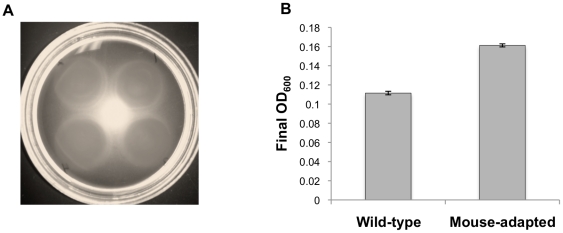
*In vitro* phenotypes of mouse-adapted *C. jejuni*. (A) Soft agar plates allow motile *C. jejuni* to spread from the center of inoculation. The ability to spread is based on flagellar motility. The top right spot is the wild-type variant and shown going clockwise are mouse-adapted variants passaged one, two, or three times through mice. All have spread an equal amount after 48 hours. (B) A higher final OD_600_ for the mouse-adapted variant indicates a decreased ability to autoagglutinate. Standard error bars are shown.

It has been reported that non-motile and non-autoagglutinating *C. jejuni* mutants are attenuated [37], and we hypothesized that these phenotypes may have changed during passage in mice. Spreading on soft agar plates was equal in the wild-type and mouse-adapted variants, indicating that both *C. jejuni* were fully motile (Figure 3a). Also, darting motility was seen in both variants when observed in wet mounts by bright field microscopy. However, the ability to autoagglutinate was slightly decreased in mouse-adapted cells (Figure 3b). Although the change was small, it was statistically significant and reproducible.

Adherence to, and invasion of, epithelial cells is likely important for *C. jejuni* pathogenesis. Thus, we hypothesized that adherence and invasion would be increased in *C. jejuni* after mouse adaptation. Young adult mouse colon (YAMC) epithelial cells were used to compare the ability of *C. jejuni* variants to infect epithelial cells in culture. Both variants were able to adhere to, and invade, YAMC cells. However, wild-type *C. jejuni* associated with YAMC epithelial cells approximately 5 times more than mouse-adapted bacteria (Table 4). Invasion of wild-type *C. jejuni* into YAMC cells was also higher than the mouse-adapted variant by approximately 10 times. Invasion was assayed using *C. jejuni* that had been passaged 1 or 3 times. The data presented in Table 4 are from *C. jejuni* passaged a single time, indicating that this change occurred rapidly. The adherence and invasion results from this *in vitro* study would suggest the mouse-adapted variant is less virulent. However, as described in the Introduction, the mouse-adapted *C. jejuni* was more virulent than wild-type *in vivo* in C57BL/6 IL-10^−/−^ mice [21].

**Table 4:** Epithelial cell interaction of *C. jejuni* variants.

	Colony Forming Units	
Variant	Associated	Internalized
Wild-type	(1.79±0.24)×10^5^	(5.53±0.83)×10^4^
Mouse-adapted^a^	(3.25±0.16)×10^4^	(4.87±2.19)×10^3^
Mouse-adapted efficiency^b^	0.18±0.03	0.09±0.04

a Mouse-adapted bacteria in this assay had been passaged through mice a single time.

b Fraction of mouse-adapted CFU relative to wild-type.

## Discussion

Bacteria generally respond to their surroundings by changes in gene activity through regulation at the level of transcription, translation, and/or posttranslation. However, genotypic variation in a closely related population of bacterial cells provides an alternative method for immediate adaptation to novel environmental conditions. Pathogenic bacteria are faced with novel, changing circumstances during the course of infection as they pass through different host microenvironments, and are met with an active immune response. It has been argued that passage through dynamic environments between and within hosts selects for highly mutable loci, or contingency genes [38], [39]. Here we show that all homopolymeric guanine tracts over seven bases long in the *C. jejuni* genome have variable length, and that this variation provides the genetic basis for adaptation during serial passage in a mouse model of campylobacteriosis. Since serial passage also leads to higher bacterial virulence in the model [21], mutability in contingency loci should be considered an integral part of *C. jejuni* pathogenesis.

We present multiple lines of evidence to support the conclusion that adaptive evolution is occurring by selection for existing contingency loci variants during serial passage. First, the frequencies of specific phases change significantly during *in vivo* passage. No open reading frame is completely fixed at any locus, but this is expected due to the high slipped-strand mutation rate reported for these sites [9], [15]. However, some contingency genes, namely *Cj0045c*, *Cj0170*, *Cj0565*, *Cj1145c*, *Cj1295*, and *Cj1429c*, appear to be under stabilizing selection for a single phase after passage, with only small populations of one base insertion and deletion mutants. This suggests that one phase is beneficial, but due to the stochasticity of slipped-strand mutation, a background population of variants is always present. As described in the Methods section, the mouse-adapted *C. jejuni* is actually a pool of re-isolated cells from five infected mice. Therefore, we can assume that the dominant phase in our re-sequence analysis was overrepresented in multiple mice from the previous passage, providing strong evidence of selection for the phase in question. Re-sequencing of re-isolated *C. jejuni* from individual mice in the future could provide insight into how animal-to-animal variation affects *C. jejuni in vivo* adaptation.

Differential transcript abundance of contingency genes due to serial passage provides more evidence for the importance of homopolymeric variation in *C. jejuni* adaptation. Based on *in vitro* and *in vivo* transcript comparisons, the only confirmed differences between wild-type and mouse-adapted variants were in ORFs associated with contingency genes. Additionally, all of the ORFs with differential expression also had altered phase frequencies, strengthening evidence that these gene phases are being selected *in vivo*. Interestingly, transcript abundance is decreased for *Cj0170*, which is mainly in a truncated, likely “OFF,” frameshifted phase after passage, while transcript abundance is increased for *Cj1295* and *Cj1296/7*, which are in extended, likely “ON,” in-frame phases after passage. It is possible that ribosome-coated, in-frame contingency gene transcripts have greater stability *in vivo*, and thus a longer half-life [40]. Also, rho-dependent transcript termination may occur during ribosome pausing when a premature stop codon is reached for frameshifted contingency genes [41]. Alternatively, it is possible that one or more contingency genes may be directly influencing transcription of other contingency loci. In what has been termed the “phasevarion,” phase variable DNA methylation by a type III restriction-modification enzyme has been shown to control transcription of a set of genes [42], [43]. Some contingency genes are controlled under the phasevarion in *Haemophilus influenzae*, but many non-contingency genes are also part of this regulon [43]. It may be that the observed increase in the extended form of *Cj0031*, encoding a putative type IIS restriction-modification enzyme, is driving transcriptional changes in a fraction of the cell population in mouse-adapted *C. jejuni*. The mechanism of differential transcript abundance in contingency genes is under investigation in our laboratory.

Finally, our conclusion that contingency genes provide the genetic basis for adaptive microevolution during serial passage is supported by the lack of other genetic changes in the genome of mouse-adapted *C. jejuni*. No SNP or DIP reached fixation after three rounds of serial passage in the mouse model, and there were no large rearrangements detectable by PFGE. The SNPs that were detectable below the level of fixation, such as the one that arose in *pbpC*, may be beneficial mutations, but are not likely driving the increased virulence that is observed. If these SNPs were the cause of increased virulence, we would expect them to become fixed after multiple rounds of passage. Also, increased virulence after a single passage is consistent with our conclusion that beneficial mutations at contingency loci are present in the original inoculum. Still, the SNPs described may be beneficial mutations that we could expect to become fixed in the population after more rounds of passage. Such a complete genome analysis is only possible through re-sequencing, and in particular, through the next-generation sequencing technologies that make comparative genome sequencing feasible. Traditional sequencing and genome assembly, or next-generation sequencing to low coverage, would not have detected any difference between wild-type and mouse-adapted *C. jejuni* due to insufficient coverage across homopolymeric tracts.

Although we have implicated thirteen contingency loci in host adaptation and enhanced virulence, the functions during pathogenesis of these altered loci are not well established. Three significant changes were in putative pseudogenes, but for *Cj0046* and *Cj0676*, passage results in an extended ORF. It is possible that this extension results in some gene product activity. Of the thirteen gene changes, only *Cj1295* has been functionally characterized [44]. The *Cj1295* gene product has been associated with a pseudaminic acid modification that is part of the *C. jejuni O*-linked flagellar glycan profile, but a role in virulence or host adaptation has not been previously described. In addition to *Cj1295*, four other putative flagellar glycosylation genes were significantly altered in mouse-adapted *C. jejuni*, suggesting flagellar modifications are important for host adaptation and enhanced virulence. Apart from flagellar glycosylation changes, putative carbohydrate-modifying contingency genes in the capsule locus and lipooligosaccharide (LOS) locus also changed significantly. Because flagella and surface structures are known stimulators of innate and adaptive immunity, we speculate that these changes may decrease recognition by host cell surface and cytoplasmic receptors allowing expansion of the *C. jejuni* population. In particular, the large decrease (-7.6 fold) of in-frame *Cj1145c*, a homolog to an LOS glycosyltransferase in *C. jejuni* LIO87 [45], may be due to immune system recognition and clearance of bacterial cells expressing the in-frame, active protein product of *Cj1145c*.

A surprising finding in this study was that the mouse-adapted bacteria appeared to be less virulent by *in vitro* assays. Mouse-adapted *C. jejuni* was less able to autoagglutinate than wild-type, but this was a very small difference. When Guerry *et al.*, correlated autoagglutination and virulence, they compared strains that showed large differences in the ability to autoagglutinate [34]. The small difference observed in this work is likely related to subtle alterations in surface carbohydrate modifications that are affected by the phase variable changes we observed by re-sequencing. The decreased ability of mouse-adapted *C. jejuni* to adhere and invade in cultured cells was unexpected since the ability to interact with gastrointestinal tract epithelial cells *in vivo* must be important for *C. jejuni* host colonization [46]. However, discordance between *in vitro* adherence and invasion results and *in vivo* virulence measures has been described before [47]. Also, screens using epithelial cell culture models have been unable to discover significant invasion factors, outside of genes affecting motility. Furthermore, it is known that different cell lines often produce mixed results [48]. It is likely that the inability to mimic complex host physiologic factors in the limited environment of the culture dish makes *in vitro* models inappropriate for studying some aspects of the molecular basis of *C. jejuni* pathogenesis.

Based on homopolymeric hypervariation, Parkhill *et al.* suggested that *C. jejuni* has quasispecies properties similar to RNA viruses such as human immunodeficiency and hepatitis C virus [9], [49], [50]. Our analysis shows that *C. jejuni* exists as a population of genotypes within a host, which is a main property of quasispecies. However, another feature of the quasispecies concept is that selection is acting on the population and not individual variants. From our data it can be seen that some contingency loci phases sweep to near fixation during passage, while others are stable as a mixed population. If selection was acting on individuals within the population, all contingency genes should be nearly fixed to one phase since they would have been selected, or hitchhiking, in the most fit genome. In our analysis it appears that selection at one locus does not have any affect on the distribution of mutations at other loci. However, this may be a consequence of the high mutation rate at homopolymeric tracts leading to the re-diversification of tract length distributions at contingency loci under no selective pressure. Also, it is possible that multiple *in vivo* microenvironments, or a variable host immune response, exert dynamic selective pressures on variants within the population to maintain genome diversity in the *in vivo* population. Based on our data and the work of Parkhill *et al*. [9], researchers should consider *C. jejuni* as a population of genotypes and phenotypes, and not a clonal isolate.

Generation of genetic diversity during *in vivo* growth has been described in *C. jejuni* [28], [29], [30], [51], but these studies mainly focused on *C. jejuni* infection of chickens. In regards to homopolymeric tract variation, Wilson *et al.* suggested that generation of genetic diversity occurs in specific, permissive environments such as the avian intestine where *C. jejuni* is a commensal organism [51]. Generation of diversity in avian species may be advantageous for transmission to novel hosts, such as different mammalian species, where diversity is lost due to stabilizing selection. In our re-sequencing study, some contingency genes had a specific phase enriched after passage, concomitant with a loss of diversity. However, in this experiment contingency gene phases are never lost from the population and sixteen genes maintained a stable distribution of variants after passage through mice. Also, the generation of homopolymeric diversity implies there are no restrictions on the mutations that arise and are maintained in these tracts. However, as suggested by Wassenaar *et al.*, some tract length frequency distributions do not appear to be stochastically maintained since the frequency of one frameshifted length is favored [15]. If a purely stochastic, slipped-strand mutational process was driving this distribution we would expect to see no bias between the insertion and deletion frameshift mutations since they both result in the same prematurely stopped protein product. As an example, for *Cj0685c,* only two homopolymeric tract lengths are observed in our analysis (in-frame and one base deletion frameshift), and this bi-allelic distribution is indistinguishable before and after mouse-adaptation. Here we show that a subset of contingency loci had changes in the frequency of homopolymeric tract lengths due to mouse passage, and we suggest that similar changes would occur in some contingency loci during avian passage.

As noted by Moxon *et al.*, contingency gene switching is combinatorial [14]. Therefore, the 29 variable regions described in *C. jejuni* may produce at least 2^29^, or ∼536 million genotypes, and a potentially equal number of phenotypes that provide a substrate for rapid adaptation. In this work we show that standing genetic variation in these genes is capable of driving the rapid adaptation of *C. jejuni in vivo*. Aside from contingency gene variations, the mouse-adapted variant has a nearly identical genome to wild-type, but with significantly different virulence properties. Therefore, it is necessary to consider the potential effects of contingency gene variability during *C. jejuni* human clinical trials, molecular epidemiology studies, and when developing successful vaccines and therapies against *Campylobacter jejuni*. Additionally, contingency loci affecting surface structures could be particularly significant from a clinical perspective when considering that outer membrane changes in *C. jejuni* have the potential to initiate autoimmune disease in the host, such as Guillain-Barré syndrome.

## Methods

### Ethics Statement

All experiments involving mice were performed according to the recommendations in the Guide for the Care and Use of Laboratory Animals of the National Institutes of Health. Protocols were reviewed and approved by the University of Michigan Committee on Use and Care of Animals (Application Number: 09116).

### Bacteria

*C. jejuni* NCTC11168 was obtained from the American Type Culture Collection (ATCC 700819), and was streaked to generate the wild-type inoculum for serial passage. The mouse-adapted variant was generated in the serial passage experiments described Bell *et al* [21]. Briefly, 5 mice were colonized with wild-type NCTC11168 for 35 days or until clinical signs necessitated early euthanasia. At necropsy, cecal tissue was streaked on selective media to re-isolate *C. jejuni* from all 5 mice. Re-isolations were pooled, frozen, and used to generate the inoculum for the next passage. The mouse-adapted variant that was re-sequenced in this study was the inoculum that was used to infect the final round of mice and had been passaged three times. Variants were generally streak-plate cultured at 37°C on Bolton or Blood agar in vented GasPak jars in a microaerobic environment generated by atmosphere evacuation, followed by equilibration with a gas mixture of 80% N_2_, 10% CO_2_, and 10% H_2_. At most, one subculture was performed prior to genotypic or phenotypic analysis.

### Illumina Sequencing and SNP analysis

The wild-type and mouse-adapted inocula were streaked onto Tryptic Soy Agar with 5% defibrinated sheep's blood (TSAB) and grown for 48 hours as described above. The entirety of the growth on streaked plates was harvested into phosphate buffered saline and pelleted before DNA extraction using the Qiagen DNeasy blood and tissue kit according to the manufacturer's instructions. Sequencing was carried out at the Michigan State University Research Technology Support Facility (MSU RTSF). DNA was prepared for sequencing using the Illumina Genomic DNA Sample Prep Kit following the manufacturer's instructions. The flow cell was generated via the Single Read Cluster Generation Kit (v. 2) and sequencing carried out on an Illumina GAIIx using the Sequencing Reagent Kit (v. 3). Data was collected with Sequencer Control Software v2.6 and base calling was performed with Real Time Analysis v1.6.

Analysis of SNP mutations in mixed bacterial genotypes was as described by Barrick *et al*. [24], [52]. Briefly, reads were mapped to the reference genome (GenBank: AL111168.1) using SSAHA2 [53]. The frequencies of all possible base mismatches and single-base indels at read bases with a given quality score were counted at reference genome positions with only uniquely aligned reads to create an empirical error model. Then, the probability of observing the aligned bases and their quality scores at every position with only uniquely aligned reads in the reference genome was calculated according to two models. The first model assumed that the entire population had only one base at this position and that all disagreements were therefore due to sequencing errors. A polymorphism was predicted when the second model that allowed the population to be an arbitrary mixture of the two most frequent bases at this position explained the data better by a likelihood ratio test (E-value ≤0.01).

Because this procedure alone has a high false-positive rate due to biases in sequencing errors that are not accounted for by base quality scores, polymorphism predictions had to pass three further tests. First, the distribution of reads with each of the two bases between the two possible genome strands had to be similar by Fisher's exact test (P-value ≥0.01). Second, the qualities of bases supporting the polymorphism had to not be biased toward lower scores compared to those supporting the reference base by a Kolmogorov-Smirnov test (P-value ≥0.05). Finally, only polymorphisms where both bases are predicted to be present at frequencies ≥5% in the population are reported.

Illumina read data has been deposited into the NCBI short-read archive (SRA023661.1).

### Contingency gene variation analysis

Reads were mapped onto the *C. jejuni* NCTC11168 reference genome (GenBank: AL111168.1) using ZOOM v1.5 Next Generation Sequencing Software [54]. Parameters were set to allow at most 2 mismatches per read with at least 8 high quality bases, and an indel length of at most 3 bases. Therefore, if the reference homopolymeric tract contained 9 bases, reads with between 6 and 12 bases in the tract could be mapped. After mapping, manual curation of indel variation in known and suspected variable regions was performed. Reads were considered informative if they satisfied two conditions. First, the read had to extend across the homopolymeric tract with a consistently high quality score throughout. Second, there had to be at least 1 high quality base before or after the tract that matched the reference sequence and signaled the end of the homopolymeric tract. The distribution of reads counted for each contingency locus can be found in Table S3. Changes in frequencies of observed homopolymeric tract lengths were considered significant at P-value <0.01 from chi-square tests of association. It was recognized that tracts with different numbers of bases in a given gene could result in the same open reading frame. When this happened, tract lengths that gave the same ORF were grouped for statistical analysis. Where possible, the longest ORF based on the homopolymeric tract lengths observed was considered to be the full-length, in-frame form of the contingency locus. Then, a fold change of the in-frame ORF was calculated by comparing the frequency that the in-frame ORF was observed in the mouse-adapted population relative to the unpassaged wild-type population.

### Colony PCR and Sanger Sequencing

A small amount of intact *C. jejuni* cells from a single colony on a streak plate were toothpicked into PCR master mix. Denaturation/lysis at 95°C, 10 minutes was done before initiation of thermocycling. PCR products were purified using the Qiaquick PCR clean-up kit (Qiagen) according to the manufacturer's instructions, and DNA sequencing was performed at the MSU RTSF.

### Pulsed Field Gel Electrophoresis

*C. jejuni* cells were harvested from Bolton agar plates, and PFGE was performed on SmaI-digested genomic DNA of each variant as described by Ribot *et al.* [55], using the Chef-DR™ system (Bio-Rad).

### Germ-free mouse experimental design for gene expression comparisons

C57BL/6 IL-10^−/−^ mice were raised in the germ-free colony at the University of Michigan (Ann Arbor). Mice were housed in soft-sided bubble isolators, and fed autoclaved water and laboratory chow. At six weeks of age, male mice were inoculated with approximately 1×10^10^ CFU of either wild-type or mouse-adapted *C. jejuni*. Preparation of the inoculum is described in Mansfield *et al.* [32]. All mice infected with a given variant were housed together in an autoclaved, polycarbonate filter-top cage inside a laminar flow hood. Infection was allowed to proceed for 96 hours before euthanasia. Mice were humanely sacrificed by giving an overdose of an inhalant anesthetic according to AVMA guidelines [56]. The cecum was immediately extracted, submerged in RNAlater (Ambion) and the cecal contents were squeezed out using a sterile cell lifter (Corning). Samples were stored in 50 ml conical tubes in RNAlater on ice for approximately 2.5 hours until they were stored at −80°C until RNA extraction. RNAlater has been used successfully with *C. jejuni* [57], and in our hands these storage conditions did not alter *C. jejuni* NCTC11168 gene expression in control microarray experiments (data not shown).

### *In vivo* RNA isolation

This protocol was modified from Zoetendal *et al.* [58]. Briefly, cecal contents in RNAlater were thawed on ice, and an equal volume of PBS plus a 1∶100 volume of phenol (Invitrogen) was added directly to each tube. Tubes were vortexed before slow-speed centrifugation, supernatant extraction, and then high-speed centrifugation. This procedure resulted in pellets that consisted of only *C. jejuni* cells, and small cecal content particles. RNA was then extracted using TRIzol® (Invitrogen) according to the manufacturer's instructions. To remove proteins, fat, polysaccharides, proteoglycans, and insoluble materials, centrifugation after homogenization, and precipitation using a high salt solution and isopropanol was performed as described in the TRIzol® reagent protocol. Samples were treated for vigorous DNA contamination with the TURBO DNA-free™ kit (Ambion) and then precipitated overnight with a 1∶10 volume of 3M sodium acetate, pH 5.5, and 3 volumes of 100% ethanol.

### *In vitro* RNA isolation

Frozen inocula were streaked onto Bolton agar plates and harvested into sterile tryptic soy broth with 15% glycerol to OD_600_ 0.3, before being aliquoted and stored at −80°C. Ten milliliters Bolton broth in 25 cm^2^ vented tissue culture flasks were inoculated with 100 µl of thawed aliquot. Cells were grown at 37°C with 80 r.p.m. agitation inside a microaerobic GasPak jar. After 18 hours, a 1∶10 volume of phenol “stop solution” [59] was added to the culture before RNA was extracted using the Qiagen RNeasy Mini kit (Qiagen) according to the manufacturer's instructions. The optional on-column DNase step was performed, and after elution samples were precipitated overnight with a 1∶10 volume of 3M sodium acetate, pH 5.5, and 3 volumes of 100% ethanol. Samples were then treated using the TURBO DNA-free™ kit.

### Microarrays and analysis

The above methods produced intact RNA as verified by analysis on the Agilent BioAnalyzer 2100, and visualization on non-denaturing TAE agarose gels. During real-time qRT-PCR, residual DNA could not be detected in control wells before 32 cycles, further verifying the lack of genomic DNA in RNA samples. RNA was reverse transcribed overnight in the presence of aminoallyl-dUTP (Ambion) using SuperScript III (Invitrogen), and cDNA was labeled with Cy3 or Cy5 dye (GE Healthcare). Whole-ORF arrays containing ∼99% of *C. jejuni* NCTC11168 ORFs were hybridized overnight in a rotating 54°C oven using microarray hybridization chambers and backing slides from Agilent. Array details can be found at the NCBI GEO (GPL8707 and GPL8954). Scanning and fluorescence data generation was done using GenePix scanner and software. LimmaGUI was used for global loess normalization of fluorescence intensities, and to determine significant expression differences [60]. Results were considered significant at p<0.05 after applying a false discovery rate control. Reports from microarray experiments are deposited in the NCBI GEO (GSM437708-437714 and GSM587271-587274) and conform to MIAME guidelines.

### Real-time quantitative reverse transcription PCR (qRT-PCR)

Primers for real-time qRT-PCR were designed using web-based Primer3 [61] to amplify 80–120 bp of template (Table S4). Reactions were carried out in 96-well PCR plates using a Bio-Rad iQ5 iCycler and iScript One-Step RT PCR Kit with SYBR Green (BioRad). Threshold cycle number was determined for four biological replicates from each variant. Relative expression was normalized to the 16S rRNA threshold cycle and analyzed by the 2^−ΔΔCt^ method described by Livak and Schmittgen [62].

### Cell culture

Young Adult Mouse Colon (YAMC) epithelial cells are described in Whitehead et al. [63] and were obtained with permission from Dr. Robert Whitehead (Ludwid Institute for Cancer Research, Melbourne, Australia). YAMC cells proliferate at 33°C in the presence of IFN-γ due to IFN-γ inducible expression of the temperature sensitive SV40 large T antigen. Cells were propagated in 5% CO_2_ at 33°C in RPMI medium 1640 with L-glutamine and 25 mM HEPES (Invitrogen), supplemented with 5% fetal bovine serum, ITS® (BD Biosciences; insulin 6.25 µg/ml, transferrin 6.25 µg/ml and selenous acid 6.25 ng/ml), 5 IU/ml of murine IFN-γ, and 100000 IU/l penicillin and 100 mg/l streptomycin (permissive conditions). Before *C. jejuni* infection, cells were moved to 5% CO_2_, 37°C and incubated without antibiotics, ITS®, or IFN-γ (non-permissive conditions). It has been reported that these cells behave similarly to normal colonic epithelial cells at the non-permissive temperature in that they are contact inhibited, produce brush border enzymes, and undergo apoptosis as they reach maximal confluence [63], [64].

### Association and invasion assays

Approximately 1.5×10^5^ YAMC cells were transferred to each well of a 24 well tissue culture plate and incubated to ∼80% confluence for 48 hours. YAMC cells were moved to 37°C infection conditions 18 hours prior to addition of *C. jejuni*. For association assays, *C. jejuni* was added to an MOI of 100∶1 and allowed to colonize for 1 hour before 3 warm PBS washes, lysis with 0.1% Triton X-100, and enumeration by serial dilution. The number of cells associated includes both adhered and invaded *C. jejuni*. Invasion assays were performed with *C. jejuni* MOI 100∶1, and following a two hour incubation, cells were washed with PBS and incubated for one hour with 250 µg/ml gentamicin to kill extracellular bacteria. YAMC cells were then washed again, lysed, and released bacteria were enumerated on agar plates. Results presented above are mean CFU ± standard deviation from triplicate wells during a single experiment. The association and invasion assays were performed twice to ensure reproducibility.

### Autoagglutination assays

Assays for autoagglutination were performed as described in Misawa and Blaser [37]. Briefly, *C. jejuni* variants grown on Bolton agar were suspended in 3–5 ml PBS, pH 7.2 in 5 ml polystyrene tubes (BD Falcon) to an equal OD_600_. Suspensions were allowed to incubate at 37°C for 24 hours before reading the OD_600_ of the top 1 ml. The ability to autoagglutinate is reflected in the decrease in OD_600_ reading from the original suspension. Samples were processed in triplicate or quadruplicate and the assay was repeated 3 separate times at different starting OD_600_ readings. Results presented above were from the assay with starting OD_600_ 1.0. All 3 independent assays showed the same statistically significant trend (i.e. the mouse-adapted bacteria having a higher final OD_600_ measure), as presented in Figure 3 above.

### Motility Assays

*C. jejuni* variants were grown on Bolton agar plates for 24–48 hours, before being harvested and resuspended in TSB to an equal OD_600_ of 0.2–0.3. Five microliter aliquots were spotted onto a Bolton agar plate containing 0.4% agar. Cells were incubated at 37°C, 10% CO_2_ for 48 hours before being photographed to observe the diameter of spreading.

### Statistics

If data from wild-type and mouse-adapted variants did not have significantly different variance by F-test, then comparisons were considered significant when p<0.05 by Student's t-test. Other statistical tests used are as described for specific assays.

## Supporting Information

Figure S1 **Estimated indel error rates.** Representative graph of estimated error rates for different base substitution and indel mutations by quality score. This graph is from the re-sequencing data for wild-type when the reference base is adenine, but in all estimations, indel error rates fall below base substitution error rates.
(PDF)

Table S1 **SNP changes during passage.** All detected SNPs that changed frequency in the population by 5% or more are shown. Verification of SNPs was through traditional sequencing of PCR products to observe a mixed peak at the relevant nucleotide position.
(XLS)

Table S2 **Consensus mutations in our NCTC11168 (ATCC 700819) culture.** These mutations were found through mapping to the reference genome sequence (GenBank AL111168.1), and analysis as described in the Methods. Consensus changes in contingency loci are not shown here, but are pictured in Figure 1.
(XLS)

Table S3 (XLS)
**Distribution of homopolymeric tract lengths.** The number of Illumina re-sequencing reads containing specific homopolymeric tract length distributions at each contingency locus. The tract lengths that yield the in-frame ORF, and the percent of in-frame and frameshifted lengths for each locus before and after passage are also listed.

Table S4 **Primers used for real-time PCR.**
(XLS)
