# Supplementary figures and images for: Standing Genetic Variation in Contingency Loci Drives the Rapid Adaptation of Campylobacter jejuni to a Novel Host
Source: PLoS One. 2011 Jan 24;6(1):e16399. doi: 10.1371/journal.pone.0016399 (PMC3025981; doi:10.1371/journal.pone.0016399)

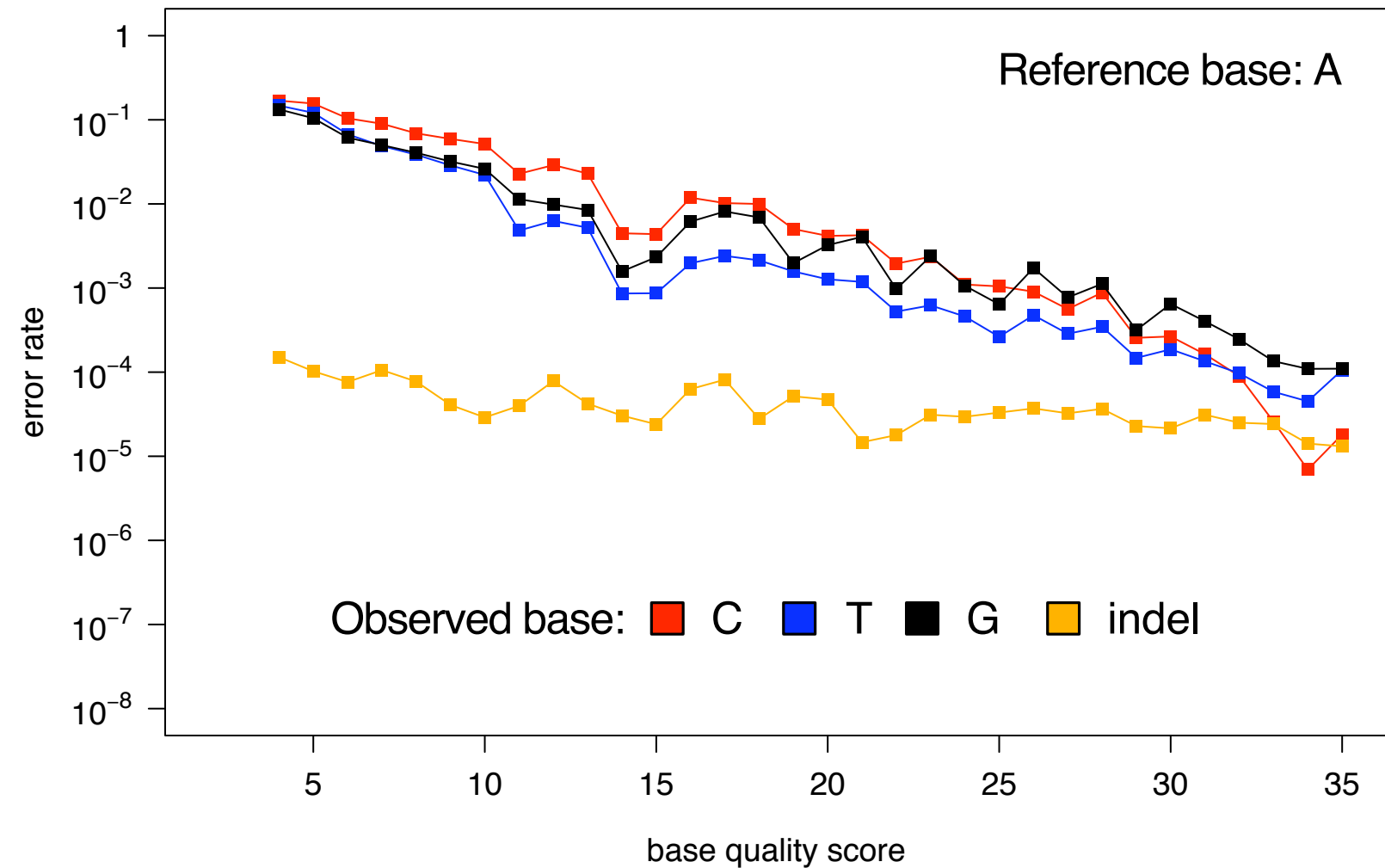

Supplement: Figure S1 — Estimated indel error rates. Representative graph of estimated error rates for different base substitution and indel mutations by quality score. This graph is from the re-sequencing data for wild-type when the reference base is adenine, but in all estimations, indel error rates fall below base substitution error rates. (PDF) [file pone.0016399.s001.pdf]
